# Systematic assessment of chemokine ligand bias at the human chemokine receptor CXCR2 indicates G protein bias over β-arrestin recruitment and receptor internalization

**DOI:** 10.1186/s12964-023-01460-2

**Published:** 2024-01-17

**Authors:** Katrijn Boon, Nathan Vanalken, Martyna Szpakowska, Andy Chevigné, Dominique Schols, Tom Van Loy

**Affiliations:** 1 Department of Microbiology, Immunology and Transplantation, Rega Institute for Medical Research, Laboratory of Virology and Chemotherapy, KU Leuven, B-3000 Leuven, Belgium; 2 Department of Infection and Immunity, Immuno-Pharmacology and Interactomics, Luxembourg Institute of Health (LIH), Esch-Sur-Alzette, Luxembourg

**Keywords:** G protein-coupled receptor, CXCR2, Chemokines, Ligand bias, NanoBRET, G protein, β-arrestin, Internalization, CXCL8, IL-8, Functional selectivity

## Abstract

**Background:**

The human CXC chemokine receptor 2 (CXCR2) is a G protein-coupled receptor (GPCR) interacting with multiple chemokines (*i.e.*, CXC chemokine ligands CXCL1-3 and CXCL5-8). It is involved in inflammatory diseases as well as cancer. Consequently, much effort is put into the identification of CXCR2 targeting drugs. Fundamental research regarding CXCR2 signaling is mainly focused on CXCL8 (IL-8), which is the first and best described high-affinity ligand for CXCR2. Much less is known about CXCR2 activation induced by other chemokines and it remains to be determined to what extent potential ligand bias exists within this signaling system. This insight might be important to unlock new opportunities in therapeutic targeting of CXCR2.

**Methods:**

Ligand binding was determined in a competition binding assay using labeled CXCL8. Activation of the ELR + chemokine-induced CXCR2 signaling pathways, including G protein activation, β-arrestin1/2 recruitment, and receptor internalization, were quantified using NanoBRET-based techniques. Ligand bias within and between these pathways was subsequently investigated by ligand bias calculations, with CXCL8 as the reference CXCR2 ligand. Statistical significance was tested through a one-way ANOVA followed by Dunnett’s multiple comparisons test.

**Results:**

All chemokines (CXCL1-3 and CXCL5-8) were able to displace CXCL8 from CXCR2 with high affinity and activated the same panel of G protein subtypes (Gα_i1_, Gα_i2_, Gα_i3_, Gα_oA_, Gα_oB_, and Gα_15_) without any statistically significant ligand bias towards any one type of G protein. Compared to CXCL8, all other chemokines were less potent in β-arrestin1 and -2 recruitment and receptor internalization while equivalently activating G proteins, indicating a G protein activation bias for CXCL1,-2,-3,-5,-6 and CXCL7. Lastly, with CXCL8 used as reference ligand, CXCL2 and CXCL6 showed ligand bias towards β-arrestin1/2 recruitment compared to receptor internalization.

**Conclusion:**

This study presents an in-depth analysis of signaling bias upon CXCR2 stimulation by its chemokine ligands. Using CXCL8 as a reference ligand for bias index calculations, no ligand bias was observed between chemokines with respect to activation of separate G proteins subtypes or recruitment of β-arrestin1/2 subtypes, respectively. However, compared to β-arrestin recruitment and receptor internalization, CXCL1-3 and CXCL5-7 were biased towards G protein activation when CXCL8 was used as reference ligand.

**Supplementary Information:**

The online version contains supplementary material available at 10.1186/s12964-023-01460-2.

## Background

The human CXC chemokine receptor type 2 (CXCR2) is a member of the chemokine G protein-coupled receptor (GPCR) family. CXCR2 is primarily expressed on endothelial cells and immune cells including neutrophils, natural killer cells, mast cells and monocytes [1]. It interacts with at least seven different chemotactic cytokines that belong to the CXC chemokine ligands (CXCLs) (i.e., CXCL1,-2,-3,-5,-6,-7,-8). These CXCR2-activating chemokines all contain the amino acid (AA) sequence Glu-Leu-Arg (ELR) preceding the conserved CXC motif and are therefore classified as ELR + chemokines. The CXCR2 signaling axis is a key mediator in immune cell migration and angiogenesis underlying both pro-inflammatory and pro-angiogenic effects [2–5]. Under pathophysiological conditions, dysregulated CXCR2 activity is associated with several inflammatory diseases as well as cancer, metastasis and chemoresistance [1, 2, 6–11].

Structurally, all GPCRs consist of an extracellular amino terminus, seven hydrophobic transmembrane (TM) segments (TMs I-VII) connected by three intra- and extracellular loops and an intracellular carboxyl terminus [12]. Activation of a GPCR leads to G protein-dependent signaling and subsequent β-arrestin recruitment as well as receptor internalization. G protein-dependent signaling involves the activation of heterotrimeric Gαβγ-proteins. Based on sequence similarity of the Gα subunit, G proteins are classified into four main families (i.e., Gα_i/o_, Gα_s_, Gα_q/11/15_ and Gα_12/13_) [12]. Each of these families is associated with subtype-specific downstream signaling. Receptor activation induces a conformational change of the heterotrimer G protein complex, enabling the exchange of GDP for GTP at the Gα-subunit and the subsequent dissociation of Gα-GTP and Gβγ subunits, both of which activate specific downstream effectors. Following this G protein activation, the receptor undergoes phosphorylation by GPCR kinases (GRKs). Depending on the phosphorylation sites, specific β-arrestin subtypes are recruited to the receptor [13, 14]. Similar to the diverse G protein subtypes, non-visual arrestins (i.e., β-arrestin1 and -2) appear to regulate different downstream signaling pathways and serve distinct physiological roles [15–17]. Overall, it is well established that both are involved in receptor desensitization and internalization, however, it has been suggested that β-arrestins might further fine-tune intracellular signaling by acting as scaffolds for various effector molecules [15–17].

Within the chemokine receptor signaling system over 20 chemokine receptors exist, which are activated by a family of about 50 chemokines [12]. Many receptors interact with multiple chemokines, and at the same time, particular chemokines serve as ligand for multiple receptors. This phenomenon was originally considered as signaling redundancy, making the chemokine signaling system highly robust and performant. Recently, this view has been challenged as it has been reported that particular chemokines are able to preferentially trigger one signaling pathway over another upon activation of the same receptor, a phenomenon referred to as ligand or agonist bias [18–20]. Although the biological relevance of such ligand bias is still poorly understood, it is conceptualized that it may be an additional mechanism for chemokine signaling finetuning [18–20]. Furthermore, in the broader context of GPCR drug discovery, interest in the existence or synthesis of receptor ligands with biased properties has emerged since this type of ligands may be endowed with improved therapeutic efficacy and/or reduced side effects [19–21].

CXCR2 is a receptor for which the potential ligand bias remains particularly unexplored. Fundamental research regarding CXCR2 signaling is rather limited and mainly focused on CXCL8 (IL-8) signaling, with CXCL8 being the first and best described high affinity ligand for CXCR2 [11]. Consequently, less is known about CXCR2 signaling and internalization induced by other chemokines that are reported to naturally interact with CXCR2 [22, 23]. Hence, it remains to be determined if all ELR + chemokines induce similar effects in terms of receptor-mediated signaling or if ligand bias exists within this signaling system. To address this question we applied a set of previously established NanoBRET-based cellular assays [24–29] to quantify the stimulating effect of the different endogenous CXCR2 chemokine ligands (*i.e.*, CXCL1-3, CXCL5-8) on G protein activation, β-arrestin1/2 recruitment and receptor internalization in a single cellular background. Based on the obtained data, ligand bias calculations were performed to quantify the existence of any bias within the CXCR2 signaling system. This analysis showed clear G protein activation bias of all ligands compared to β-arrestin recruitment and receptor internalization, when CXCL8 was used as the reference ligand.

## Methods

### Chemokines, reagents and plasmids

Recombinant human CXCL1-3 and CXCL5-8 were obtained from PeproTech. Alexa fluor 647-labeled human CXCL8 (CXCL8^AF647^) was purchased from Almac. hCXCR2 plasmid (#CXCR200000) was purchased from cDNA Resource Centre, which uses the pcDNA3.1(+) backbone. Nano-Glo® Vivazine™ substrate (#N2581) was purchased from Promega. The CXCR1/2 antagonist navarixin (#HY-10198/CS-0609) was purchased from MedChemExpress. The REGA-SIGN plasmids as well as the NanoLux plasmids (i.e., CXCR2.mNeongreen (mNG), CXCR2.NanoLuciferase (NLuc), FYVE.mNG, β-arrestin1.NLuc and β-arrestin2.NLuc) were previously described [28, 29].

### Cell lines

Human embryonic kidney cells (HEK293A) stably expressing hCXCR2 (HEK293A.CXCR2) were generated by transfecting HEK293A cells with pcDNA3.1(+) hCXCR2. hCXCR2 expression was confirmed by flow cytometry with phycoerythrin-(PE) labeled mouse anti-human CXCR2 monoclonal antibody (mAb) (Clone 5E8; Biolegend) and PE-labeled IgG1, κ isotype control mAb (clone MPOC-21; BD Pharmingen™).

Non-transfected HEK293A cells were cultured in Dulbecco’s Modified Eagle Medium, high glucose (DMEM; #41965, Thermo Fisher Scientific) supplemented with 10% Fetal Bovine Serum (FBS; #10270106, Thermo Fisher Scientific), from here on referred to as growth medium. hCXCR2-expressing HEK293A cells were cultured in growth medium supplemented with 500 μg/mL Geneticin (#10131, Thermo Fisher Scientific).

### Cell transfection and seeding

For NanoBRET assays, cells were transfected in suspension using FuGENE® HD Transfection Reagent (#E2311; Promega) at a 3:1 reagent to DNA ratio, and containing 1 μg/μL DNA. The FuGENE® HD/DNA mixture was incubated for 10 min at ambient temperature before adding it to the cell suspension. Transfected cells were immediately seeded at a density of 3.0 × 10^4^ cells/well in white, clear flat-bottom 96-well plates coated with 100 μg/mL poly-D-lysin and incubated for 48 h at 37°C and 5% CO_2_.

###  CXCL8^AF647^ saturation binding assay

HEK293A.CXCR2 and HEK293A cells were detached using 0.25% Trypsin-EDTA (Thermo Fisher Scientific, #25200056), resuspended in growth medium and incubated at room temperature for two hours. Cells were washed and resuspended in assay buffer [Hank’s Balanced Salt Solution (HBSS; Thermo Fisher Scientific), 20 mM HEPES buffer (Thermo Fischer Scientific), 0.5% FBS]. Cells (~ 1.5*10^5^ cells/well) were incubated with serially diluted CXCL8^AF647^ (2000 nM in 1:2 dilution steps) for 30 min at room temperature (RT) protected from light. Cells were then washed twice in assay buffer and fixed in Dulbecco’s phosphate buffered saline (DPBS) with 1% formaldehyde (Merck Millipore, Burlington, MA, USA). Samples were immediately processed by flow cytometry (BD FACSCelesta™ HTS, BD Bioscience, San Jose, USA). Data were analyzed with the FlowJo™ software (Ashland, Oregon, USA). Mean fluorescent intensity (MFI) and Robust standard deviation (rSD) were used to calculate the stain index (SI) [SI = (MFI_sample_-MFI_NC_)/(2*rSD_NC_)); with NC = negative control (unstained cells)]. Afterwards the SI based concentration-response curves were fitted with *One Site- Total and non-specific binding* in GraphPad V9.3.1 (GraphPad Software, San Diego, CA, USA) to obtain the Kd value.

###  CXCL8^AF647^ competition binding assay

HEK293A.CXCR2 cells were detached using 0.25% Trypsin-EDTA (Thermo Fisher Scientific, #25200056), resuspended in growth medium and incubated at room temperature for two hours. Chemokines (CXCL1-3 and CXCL5-8 or vehicle) were serially diluted in assay buffer at a final volume of 100 μL/well. Then 50 μL/well of cells (~ 1.5*10^5^ cells/well) and 50 μL/well CXCL8^AF647^ (final concentration of 25 ng/mL) was added and the mixture was incubated for 30 min at room temperature (RT) protected from light. Cells were washed and processed as described for the titration experiment. To obtain the pKi value, the SI based concentration-response curves were fitted to *One site – Fit Ki* in GraphPad V9.3.1 (GraphPad Software, San Diego, CA, USA) using the Kd value obtained in the CXCL8^AF647^ titration experiment. Statistical analysis was done with one-way ANOVA followed by a Dunnet’s test.

### NanoBRET G protein activation assay

HEK293A.CXCR2 cells were transiently co-transfected with plasmids encoding Gα protein tagged with NLuc (donor) and Gγ protein tagged with LSS-mKATE2 (acceptor) in a 1:10 donor-acceptor ratio [29]. Forty-eight hours after transfection and seeding, cells were washed with assay buffer and incubated with 90 μL of a 1:100 Nano-Glo® Vivazine™ working solution (#N2581, Promega) for 45 min at 37°C and 5% CO_2_. If navarixin was added, it was dissolved in the Vivazine solution at a concentration of 1 μM. The plate was transferred to the FLIPR Penta (Molecular Devices). After 15 min of equilibration time, baseline BRET signals were determined by five consecutive measurements immediately followed by the automatic addition of 10 μL of 10 × ligand to the cell plate by the FLIPR Penta. Changes in BRET ratios were monitored in real-time (every 2.5 s) for 25 min. Measurements were acquired using a 440-480 nm donor emission filter (#0200–6179, Molecular Devices) and a custom 615 nm AT600lp acceptor emission filter (#296420, Chroma).

### NanoBRET β-arrestin1/2 recruitment assay

HEK293A cells were transiently co-transfected with a donor plasmid encoding β-arrestin1 or -2 tagged with NLuc (β-arrestin1.NLuc or β-arrestin2.NLuc, respectively) and the acceptor plasmid encoding CXCR2 tagged with mNeongreen (CXCR2.mNG) in a 1:10 donor-acceptor ratio [28], seeded and incubated for 48 h as described. Subsequent addition of substrate (Nano-Glo® Vivazine™) and BRET reading protocol are identical as for the NanoBRET G protein activation assay, with the only difference being the use of a different acceptor emission filter (515 to 575 nm; #0200-6203, Molecular Devices).

### NanoBRET internalization assay

Receptor internalization was monitored through detection of its delivery to early endosomes. HEK293A cells were transiently co-transfected with a donor plasmid encoding CXCR2 tagged with NLuc (CXCR2.NLuc) and an acceptor plasmid encoding FYVE, a phosphatidylinositol 3-phosphate (PI3P) binding motive of endofin protein, present on early endosomes, tagged with mNG (FYVE.mNG) in a 1:100 donor-acceptor ratio [28]. Subsequent substrate (Nano-Glo® Vivazine™) addition and BRET reading protocol was identical as described for NanoBRET β-arrestin1/2 recruitment assay.

### BRET data analysis and ligand bias calculation

G protein activation, β-arrestin recruitment and receptor internalization in the BRET assays were calculated using the equations presented in *Calculation 1.* BRET ratios are the ratio of acceptor emission to NLuc donor emission. The basal BRET ratio (BRET_basal_) was defined as the mean BRET ratio of five consecutive readings prior to ligand addition. To quantify ligand-induced changes, ∆BRET was calculated for each well as a % difference to baseline. Subsequently, ∆BRET values were background corrected by subtracting the averaged ∆BRET from the negative control (NC). Eventually, the negative area under the curve (neg AUC) was used as the read-out for G protein activation, while the positive area under the curve (pos AUC) was used as the read-out for β-arrestin recruitment and receptor internalization. Concentration-response curves were fitted to *log (agonist) vs. response (three parameters)* in GraphPad V9.5.1 (GraphPad Software, San Diego, CA, USA) whereby the bottom was constrained to 0. The calculated top value was taken as E_max_. These E_max_ values were normalized relative to the E_max_ of CXCL8, which was set at 100%. Statistical analysis was accomplished with one-way ANOVA followed by a Dunnet’s test or a two-way ANOVA in case navarixin was added.

**Table:** 

***Calculation 1: Calculation of BRET signal***
1. $$BRET~ratio= \frac{{acceptor}_{em}}{{donor}_{em}}$$
2. $$\Delta BRET=\left[\frac{{BRET}_{stim}-{BRET}_{basal}}{{BRET}_{basal}}\right]\times 100$$
3. $$NC~corrected~\Delta BRET= {\Delta BRET}_{exp}-{mean~\Delta BRET}_{NC}$$
4. $$Neg~AUC\ of\ NC~corrected~\Delta BRET =G~protein~activation$$
5. $$Pos~AUC\ of\ NC~corrected~\Delta BRET =\beta-arrestin~recruitment$$

Bias index was calculated using the equations presented in *Calculation 2*. First, log (E_max_/EC_50_) was calculated using E_max_ and potency values expressed in molar (M) for each ligand in each G protein activation pathway, β-arrestin recruitment pathway and receptor internalization. Secondly, for each pathway, ∆log (E_max_/EC_50_) values are calculated by subtracting the log (E_max_/EC_50_) of the reference ligand (CXCL8) from that of the tested ligand. Finally, bias index is calculated by subtracting the ∆log (E_max_/EC_50_) of the tested ligand from the stated reference pathway from that of the pathway of interest. Statistical analysis was accomplished with one-way ANOVA followed by a Dunnet’s test.

**Table:** 

***Calculation 2: Calculation of bias index***
1. $${\text{log}}{\left(\frac{{E}_{max}}{{EC}_{50}}\right)}_{lig}$$
2. $$\Delta {\text{log}}\left(\frac{{{\text{E}}}_{max}}{{EC}_{50}}\right) ={\text{log}}{\left(\frac{{E}_{max}}{{EC}_{50}}\right)}_{lig}-\mathrm{ log}{\left(\frac{{E}_{max}}{{EC}_{50}}\right)}_{ref:CXCL8}$$
3. $$Bias\ index={\Delta {\text{log}}\left(\frac{{{\text{E}}}_{max}}{{EC}_{50}}\right)}_{P1}- {\Delta {\text{log}}\left(\frac{{{\text{E}}}_{max}}{{EC}_{50}}\right)}_{P2}$$

## Results

### ELR+ chemokines display high binding affinity to CXCR2

**Fig. 1:**
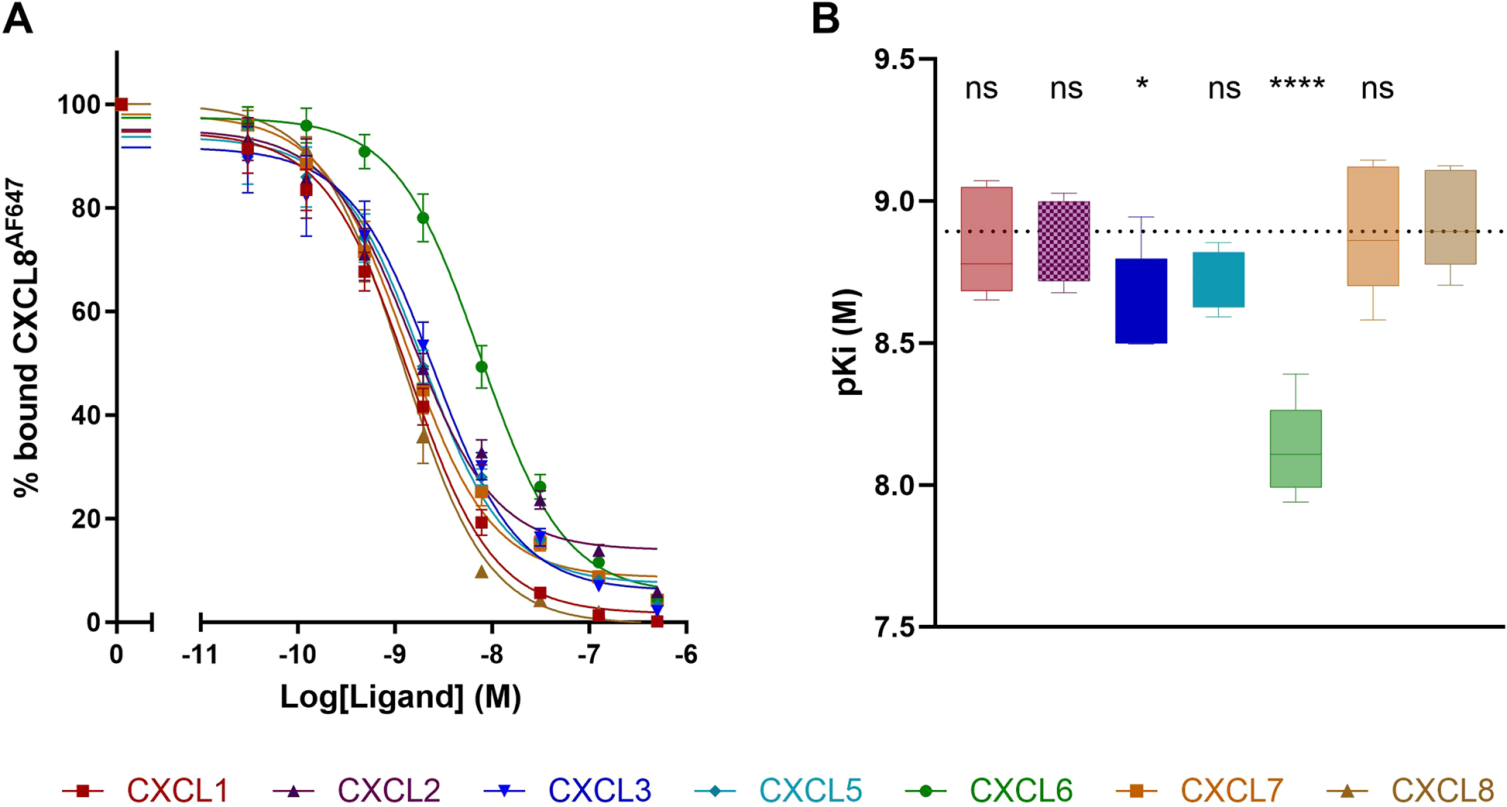
Competition binding assay shows that all ELR + chemokines are high-affinity ligands for CXCR2. A competition binding assay was performed on HEK293A.CXCR2 cells incubated with CXCL8^AF647^ (25 ng/mL) and increasing concentrations of unlabeled CXCL1-3 and CXCL5-8 as competitors. **A** Concentration-response curves showing CXCL8^AF647^ binding inhibition by unlabeled chemokines. Data are normalized using a vehicle control and show the Mean ± SEM (*n* = 5). **B** To determine the pKi values, concentration-response curves were fitted to *one site – Fit Ki* in GraphPad V9.3.1 (GraphPad Software, San Diego, CA, USA). Statistical analysis of the pKi values was performed using one-way ANOVA followed by a Dunnet’s test with CXCL8 as the reference ligand (pKi of CXCL8 is represented by the dashed line). Statistical significance is marked as **** (*P* < 0.0001) and *(*P* < 0.1), while non-significant differences are marked as ‘ns’

Prior to the investigation of CXCR2-mediated G protein activation, β-arrestin recruitment and receptor internalization, we first validated the binding affinity of all CXCR2-binding chemokines (*i.e.*, CXCL1-3 and CXCL5-8). Therefore, we conducted a competition binding assay utilizing Alexa Fluor 647-labeled human CXCL8 (CXCL8^AF647^). The resulting pKi values were determined using the Kd value of 227 nM for CXCL8^AF647^, as established through a CXCL8^AF647^ titration experiment (Figure S1). All unlabeled chemokines competed with CXCL8^AF647^ for CXCR2 binding in a concentration-dependent manner (Fig. 1A) with nearly equivalent pKi values (Fig. 1B). Notably, CXCL3 and CXCL6 had a significantly lower pKi value, indicating a lower binding affinity. Still, all seven chemokines tested in our assay were able to completely displace CXCL8^AF647^ binding with high affinity.

### ELR+ chemokines activate Gα_i1_, Gα_i2_, Gα_i3_, Gα_oA_, Gα_oB_, and Gα_15_upon CXCR2 stimulation with similar potency and efficacy

**Fig. 2:**
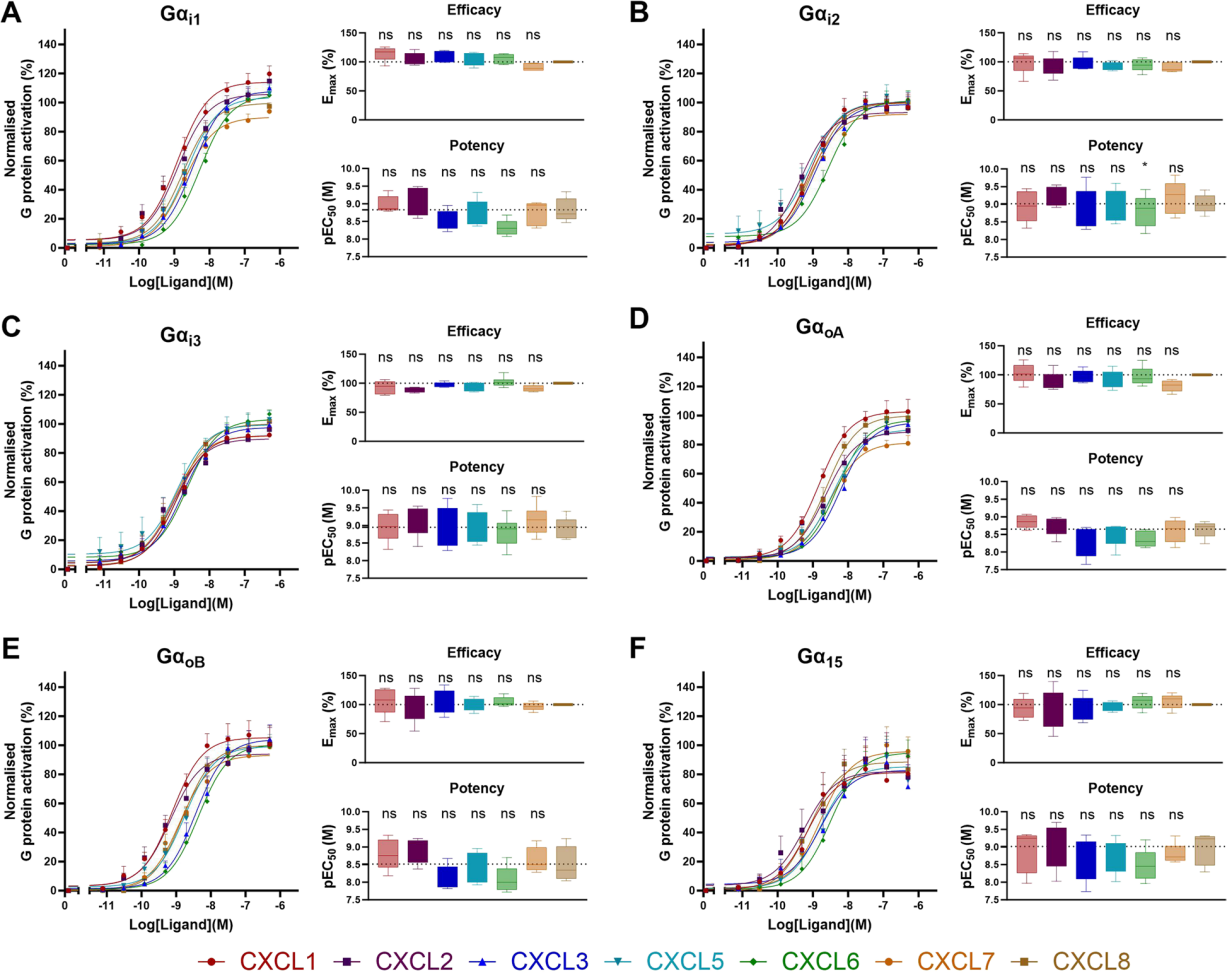
CXCR2-mediated G protein activation profile. Chemokine (CXCL1,-2,-3,-5,-6,-7 and CXCL8) concentration-dependent BRET signals measured in HEK293A.CXCR2 cells transfected with the respective G protein biosensors. Results are expressed as % of G protein activation relative to the CXCL8 maximal response set at 100%. Gα_i1_, Gα_i2_, Gα_i3_, Gα_oA_, Gα_oB_, and Gα_15_ activation data is shown in panels **A** to **F**, respectively. Data of the curves represents Mean ± SEM, while the boxplot depicts the range from minimum to maximum, with a central line indicating the median across five to six independent experiments. Concentration-response curves were fitted to *log (agonist) vs. response (three parameter)* model in GraphPad V9.3.1 (GraphPad Software, San Diego, CA, USA). Statistical analysis used one-way ANOVA with Dunnet's test (*P < 0.05, ns for non-significant differences), and dashed lines indicate the mean value of CXCL8 for reference

To investigate which G protein subtypes are activated by CXCR2 upon binding of CXCL1-3 and CXCL5-8, we performed real-time concentration-dependent BRET measurements. The previously described REGA-SIGN biosensors [29] were used to monitor the activation of heterotrimeric G proteins in HEK293A.CXCR2 cells. When testing activation of the Gα_q_, Gα_12_, Gα_13,_ Gα_sS_ and Gα_sL_ subtypes, the ELR + chemokines did not induce a BRET response (data not shown), indicating no activation of these G proteins. In contrast, for Gα_i1_, Gα_i2_, Gα_i3_, Gα_oA_, Gα_oB_, and Gα_15_ (Fig. 2A, B, C, D, E and F, respectively) concentration-dependent BRET responses were measured and potency and efficacy of the diverse chemokines with regard to G protein activation was determined by non-linear curve fitting. Given that CXCL8 is the best-described and most-studied endogenous CXCR2 ligand, it was used as reference ligand for further analyses of the obtained BRET-responses. When comparing the G protein activation induced by the chemokine ligands with the activity of CXCL8, no significant differences in terms of potency (pEC_50_) nor efficacy (E_max_) were revealed within one G protein subtype.

Additionally, to ensure CXCR2 specificity of the observed G protein activation profile, cells were pre-incubated with navarixin, a small molecule antagonist that binds to an intracellular allosteric binding pocket thereby stabilizing the inactive conformation of CXCR2 [30–33]. The presence of navarixin (1 μM) abolished the decreasing BRET signals when cells were stimulated with a fixed concentration (i.e., their respective EC_80_) of the CXCR2 ligands (Figure S2), confirming CXCR2-specificity of the signal.

### Reduced potency of ELR + chemokines in β-arrestin recruitment compared to CXCL8

**Fig. 3:**
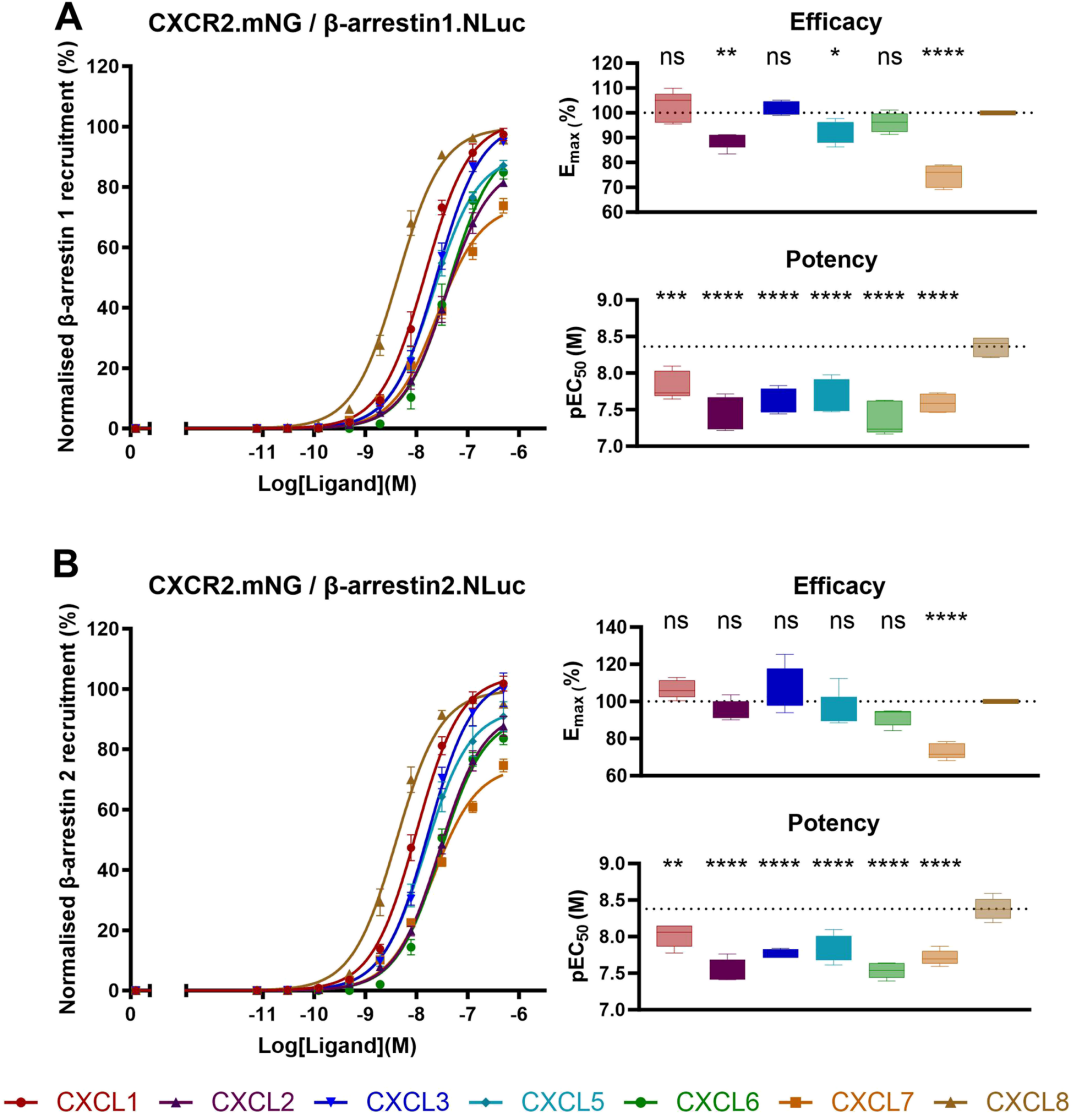
CXCR2-mediated β-arrestin1/2 recruitment. Concentration-dependent BRET signals were measured upon stimulation of HEK293A cells transfected with CXCR2.mNG and β-arrestin1.NLuc (Panel **A**) or β-arrestin2.NLuc (Panel **B**), respectively. Results are presented as a percentage of β-arrestin recruitment, with the maximal CXCL8-induced response set at 100%. Data for the curves represents the Mean ± SEM, while the boxplot depicts the range from minimum to maximum, with a central line indicating the median across five independent experiments. Concentration-response curves were fitted to *log (agonist) vs. response (three parameter)* in GraphPad V9.3.1 (GraphPad Software, San Diego, CA, USA). Statistical analysis was done using one-way ANOVA followed by a Dunnet’s test. Significant differences were marked with *(*P* < 0.05), **(*P* < 0.01), ***(*P* < 0.01) or ****(*P* < 0.0001), non-significant differences were marked as ns. The dashed lines represent the mean value of CXCL8

A previously described NanoBRET-based technique [28] was used to examine the β-arrestin recruitment mediated by CXCR2 upon binding of CXCL1-3 and CXCL5-8. HEK293A cells transfected with β-arrestin1.NLuc or β-arrestin2.NLuc and CXCR2.mNG were stimulated with increasing concentrations of chemokine and the BRET signal was measured. Unlike with the G protein activation profile, pronounced differences in potency and efficacy between the different chemokines were found (Fig. 3). Specifically, all ligands had a significantly lower potency for both β-arrestin1/2 recruitment compared to CXCL8. In case of recruitment of β-arrestin1 CXCL2, -5 and -7 had a significantly lower efficacy compared to CXCL8 (Fig. 3A) while for β-arrestin2 recruitment this was only true for CXCL7 (Fig. 3B).

### Reduced potency and efficacy of ELR + chemokine receptor internalization compared to CXCL8

**Fig. 4:**
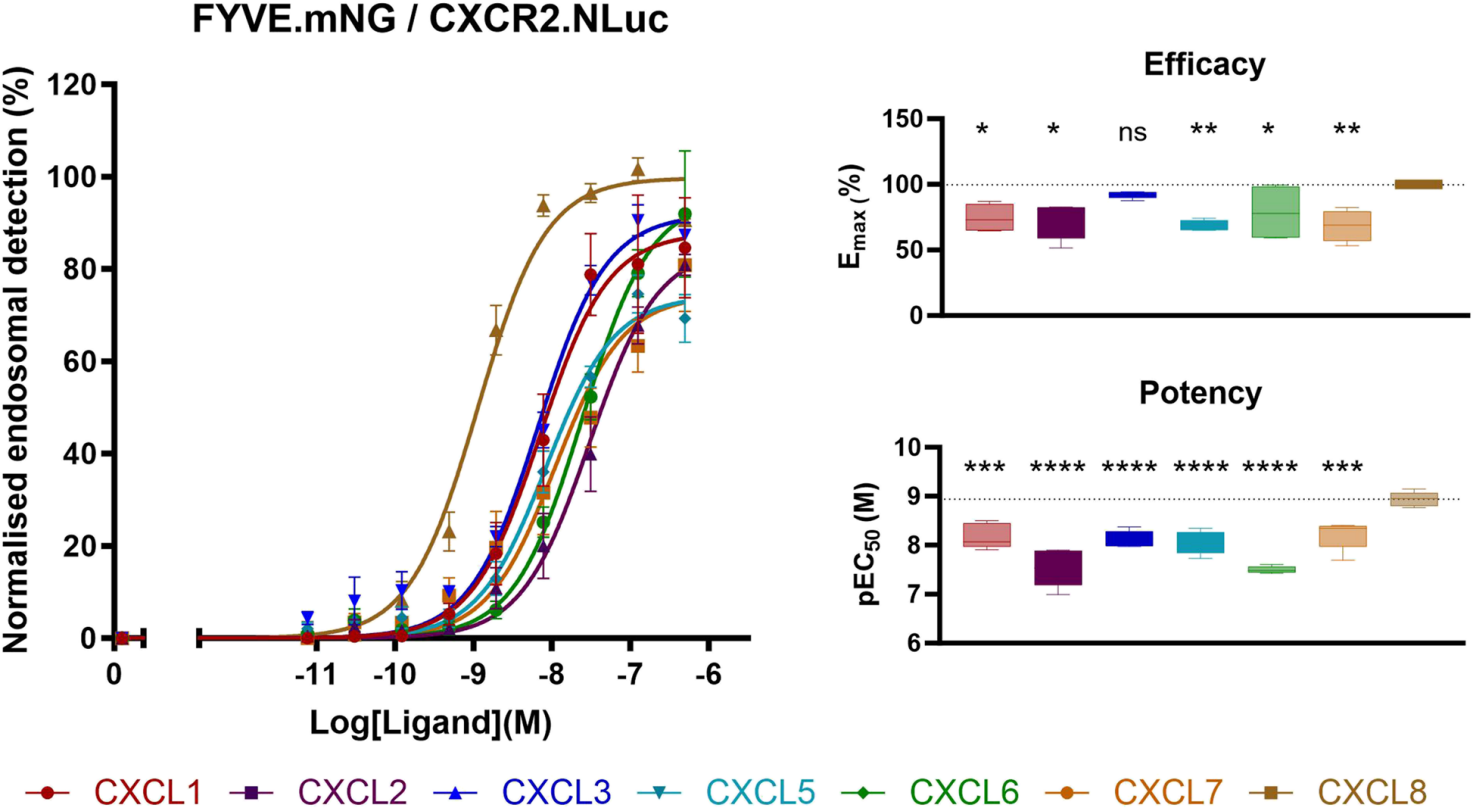
CXCR2 early endosomal trafficking. BRET signals were measured upon stimulation with increasing concentrations of CXCL1,-2,-3,-5,-6,-7 or CXCL8 of HEK293A cells transfected with the CXCR2.NLuc and FYVE.mNG. Results are expressed as % of endosomal detection whereby the maximal stimulation induced by CXCL8 was set as 100%. Data for the curves represents the Mean ± SEM, while the boxplot depicts the range from minimum to maximum, with a central line indicating the median across five independent experiments. Concentration-response curves were fitted to *log (agonist) vs. response (three parameter)* in GraphPad V9.3.1 (GraphPad Software, San Diego, CA, USA). Statistical analysis was done using one-way ANOVA followed by a Dunnet’s test. Significant differences were marked with *(*P* < 0.1), **(*P* < 0.01), ***(*P* < 0.001), or ****(*P* < 0.0001), non-significant differences were marked as ns. The dashed lines represent the mean value of CXCL8

Finally, we investigated CXCR2 internalization upon binding of CXCL1-3 and CXCL5-8. To do so, CXCR2 translocation to the early endosomes was followed using a previously described NanoBRET assay [28, 34–36] relying on the energy transfer between CXCR2.NLuc and FYVE.mNG. As shown in Fig. 4, all ligands induced concentration-dependent receptor internalization, but with significantly reduced efficacy (with exception of CXCL3) and potency compared to CXCL8.

### CXCL1-3 and CXCL5-7 exhibit G protein activation bias relative to β-arrestin recruitment and receptor internalization when CXCL8 is used as the reference ligand

The data from the G protein subtype activation assay, β-arrestin1/2 recruitment and CXCR2 internalization assay were combined to perform an in-depth quantitative analysis of ligand bias according to ΔΔlog (E_max_/EC_50_), a method previously described for other chemokine receptors [19, 26, 37]. We first aimed to explore ligand bias *within* distinct signaling pathways by comparing the activation of the different G protein subtypes by the ELR + chemokines as well as the activation of both β-arrestin subtypes. We then also explored potential ligand bias *between* signaling pathways by comparing either G protein activation vs β-arrestin recruitment, G protein activation vs receptor internalization and β-arrestin recruitment vs receptor internalization. The bias index was calculated according to *Calculation 2* whereby CXCL8 was chosen as the reference chemokine ligand, thus assuming that all pathways are activated by CXCL8 in a balanced way.

**Fig. 5:**
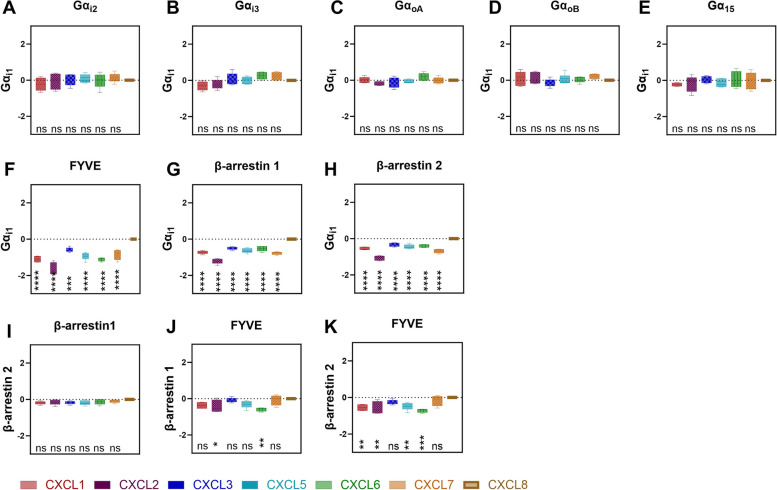
Chemokine bias index at CXCR2. Bias indexes for each chemokine between different pathway*s* were calculated using *Calculation 2* using CXCL8 as the reference chemokine. The data are represented as boxplots, which depicts the range from minimum to maximum, with a central line indicating the median across five independent experiments. Significant differences were marked with *(*P* < 0.05), **(*P* < 0.01), ***(*P* < 0.001) or ****(*P* < 0.0001), non-significant differences were marked as ns. The dashed line represents the mean value of CXCL8, being 0

First, we calculated if upon CXCR2 activation one particular Gα subtype is differently activated by a particular chemokine compared to the other Gα subtypes [26, 38] i.e. if there is chemokine ligand bias between the Gα-subtype specific pathways. As Gα_i1_ was the first subtype to be studied for G protein signaling bias [25, 27, 38], we chose to determine the bias index by comparing the activation of all G protein subtypes to the activation of Gα_i1_. As shown in Fig. 5 A-E, all bias indexes ranged around 0, indicating no or only weak bias and thus the absence of significant ligand bias in terms of G protein activation, when compared to CXCL8-mediated Gα_i1_ activity. Similarly, we examined the potential presence of ligand bias within the β-arrestin subtypes (i.e., β-arrestin1 and -2). Also here, no apparent ligand bias was identified (Fig. 5I). We also examined if ligand bias was present between β-arrestin1/2 recruitment and Gα-protein activation. Gα_i1_ was taken as reference for the G protein activation pathway. It is clear that all ligands are significantly more prone to activate G protein signaling compared to the β-arrestin signaling pathways or receptor internalization (Fig. 5F-H). Consequently, they are considered as G protein biased ligands. Finally, when evaluating ligand bias between β-arrestin activation and receptor internalization (Fig. 5J-K) CXCL1,-2,-5 and CXCL6 showed β-arrestin bias as they favored β-arrestin2 activation compared to receptor internalization. For β-arrestin1 the same trend could be observed, but bias towards β-arrestin1 was only statistically significant for CXCL2 and CXCL6.

## Discussion

Many human chemokine GPCRs can interact with multiple chemokine ligands, albeit sometimes with varying binding affinity. This phenomenon contributes to chemokine signaling being a complex network with many promiscuous ligand-receptor interactions that regulate immune cell activation and migration. The human chemokine receptor CXCR2 is a notorious example of this complexity given that it is able to interact with seven different chemokines, which might suggest signaling redundancy, or differences in spatiotemporal expression of the respective chemokines throughout development and different (patho-)physiological conditions [39, 40]. At the level of CXCR2 activation, however, it also raises the question whether all these different ligands induce similar receptor-mediated signaling events, or alternatively, display differences in their preference for activating particular signaling pathways or intracellular effector molecules. This latter scenario would be defined as ligand bias. To our knowledge, no systematic assessment of ligand bias, in terms of direct activation of G proteins, β-arrestin recruitment and receptor internalization, has so far been performed for the CXCR2 signaling system. Therefore, in this study we analyzed the activity of all the ELR + chemokines upon CXCR2 stimulation using a panel of NanoBRET-based assays that directly (i.e., without signal amplification) measure G protein activity, β-arrestin1/2 recruitment and receptor internalization.

First, competition binding experiments confirmed that the seven ELR + chemokines included in this study (*i.e.*, CXCL1,-2,-3,-5,-6,-7,-8) show high affinity for CXCR2, with only CXCL3 and CXCL6 having a significant lower binding affinity compared to CXCL8. It should be noted that with this binding assay CXCL8^AF647^ displacement was assessed, with CXCL8 being the best-described high-affinity CXCR2 ligand. It has been documented that diverse ELR + chemokines (*e.g.*, CXCL1, CXCL7) interact with CXCR2 via distinct AA residues in the receptor binding pocket [22, 41], suggesting that their binding mode does not exactly overlap with each other and with the binding mode of CXCL8. Such potential differences in binding epitopes might also (partially) explain the somewhat lower binding affinity observed for CXCL3 and CXCL6 in our competition binding assay.

We quantified G protein activation, β-arrestin1/2 recruitment and receptor internalization induced by the ELR + chemokines using previously established NanoBRET-based cellular assays [24–29]. The REGA-SIGN biosensors [29] were used to evaluate CXCR2-mediated G protein activation (Fig. 2). Our findings align well with previously reported signal transduction studies showing a similar G protein activation profile for all ELR + chemokines (i.e., signaling through Gα_i1_, Gα_i2_, Gα_i3_, Gα_oA_, Gα_oB_, and Gα_15_) compared to CXCL8-mediated CXCR2 activation. These previous studies only included CXCL8, but employed both downstream second messenger assays and BRET-based G protein effector membrane translocation studies [22, 42, 43], suggesting that BRET-based biosensors like REGA-SIGN are indicative of downstream signaling pathways. CXCR2-mediated β-arrestin1/2 recruitment and receptor internalization were quantified using the NanoLux biosensors [28]. Whereas for G protein activation no qualitative nor quantitative differences were observed (i.e., all chemokines induced activation of the same G protein subtypes with similar potency and efficacy) CXCL8 displayed significantly higher potency in β-arrestin1/2 recruitment and receptor internalization compared to other chemokines (Figs. 3 and 4). CXCL7 showed significantly less efficacy in β-arrestin recruitment, and, notably, all chemokines (except CXCL3) exhibited reduced efficacy in receptor internalization compared to CXCL8. Importantly, this diminished efficacy and potency was not observed in receptor binding or G protein activation assays. These findings suggest a preference for G protein activation over β-arrestin recruitment in the case of CXCL7, and a preference for G protein activation over receptor internalization for all chemokines except CXCL3 when CXCL8 was used as reference ligand. Additionally, in the investigation of CXCL2 and CXCL5 responses in the β-arrestin recruitment assays (Fig. 3), a significant reduction in potency was accompanied by a decrease in efficacy when activating β-arrestin1, while efficacy for β-arrestin2 remained relatively unaffected. This contrast suggests potential ligand bias between β-arrestin1 and β-arrestin2, indicating distinct downstream signaling pathways associated with these two arrestin subtypes [15–17].

To address the potential ligand bias systematically, we performed ligand bias calculations (Fig. 5). In the literature, various methods for calculating ligand bias have been proposed, with two of the most commonly described approaches being ΔΔlog (E_max_/EC_50_) and ΔΔlog(τ/KA). The latter method is often considered more accurate because it takes into account receptor density and coupling within the assay system. However, in our specific case, where concentration-response curve factors are calculated using a Hill slope of one, the values obtained from both techniques are expected to be equivalent. Hence, as it is also the most applied method, we chose to use ΔΔlog (E_max_/EC_50_), which involves comparing the log ratio of E_max_ and EC_50_ values for two ligands and subsequently for two pathways [37]. All methods for ligand bias calculations require the use of a reference ligand, which is considered to activate all signaling pathways in a balanced way. Such reference ligand is chosen arbitrarily and often is the ligand that has been most extensively studied and for which the most information is available. In our case we chose CXCL8 to be the reference ligand. One needs to bear in mind that if the reference ligand is biased itself, all ligands behaving in a same biased manner will not be detected as biased ligands [26, 37]. Nevertheless, our data indicate that all ELR + chemokines activate G protein subtypes in a well-balanced manner without any significant ligand bias (Fig. 5A-E).

Ligand bias may not only manifest itself between diverse G protein activation pathways, but can also exist between categories of GPCR transducers as well. We therefore expanded our analysis to quantify ligand bias of ELR + chemokines comparing G protein activation, β-arrestin1/2 recruitment and receptor internalization. Compared to β-arrestin1/2 recruitment, we were able to confirm that all the ligands displayed significant bias toward G protein activation relative to reference chemokine CXCL8 (Fig. 5G-H, E). Notably, a previous study [19] including all ELR + chemokines but CXCL7, looked into CXCR2 ligand bias between G proteins and β-arrestin using CXCL1 as the reference ligand. They concluded that CXCL8 was β-arrestin biased compared to CXCL1, confirming our findings as we show that CXCL1 is G protein biased compared to CXCL8. Interestingly, the only other bias found compared to CXCL1 was CXCL6 being G protein biased. Although they did not include CXCL7 and used assays more downstream of the signaling cascade making them prone to signal amplification errors, it gives an important side note that ligand bias is always relative to the reference ligand and one should be careful how to interpret it.

It is important to acknowledge that the physiological relevance of any bias detected in our study remains to be fully elucidated. Furthermore, it is worth noting that CXCR2 exists in several isoforms, some of which have been associated with hematological traits and diseases [22, 44–46]. Likewise, with respect to CXCL8 signaling, it is well-documented that post-translational modifications can significantly alter its internalization and signaling properties for both CXCR1 and CXCR2 [47]. In our study, we focused on the full-length isoform of CXCR2 as well as a single recombinant protein for each chemokine. Expanding the investigation to include various isoforms could add another layer into the intricate signaling dynamics associated with CXCR2. Nonetheless, throughout the experiments, we minimized the potential influence of system bias by conducting all assays within a consistent HEK293A cellular context. Additionally, our choice of read-out methods were not subjected to signal amplification, thereby enhancing the reliability of bias index calculations [37]. These efforts toward maintaining consistency have established a crucial foundation for unraveling the complexities of signaling bias associated with CXCR2.

## Conclusion

This study presents an in-depth analysis of signaling bias upon CXCR2 stimulation including all its ligands, i.e., the ELR + chemokines CXCL1-3 and CXCL5-8. Although no bias was identified within G protein or β-arrestin subtype activation, a distinct ligand bias favoring G protein activation over β-arrestin recruitment and GPCR internalization was evident, when CXCL8 served as the reference ligand. Interpreting this ligand bias should be done with care as an alternative choice of reference ligand might alter the outcome. Nonetheless, documenting this bias at the level of receptor activation is a first crucial step in understanding why CXCR2 interacts with seven different ligands.

### Supplementary Information

**Additional file 1: Figure S1. **CXCL8^AF647^ titration. **Figure S2.** G protein activation by CXCR2 upon stimulation with its endogenous chemokine ligands pre-treated with CXCR2 inhibitor or vehicle.

## Data Availability

All data generated or analyzed during this study are included in this published article and its additional files.

## Abbreviations

AA: Amino Acid
BRET: Bioluminescence Resonance Energy Transfer
CXCL1-8: CXC chemokine ligand 1-8
CXCL8^AF647^: Alexa Fluor 647-labeled human CXCL8
CXCR2: CXC chemokine receptor 2
NLuc: Nanoluciferase
mNG: mNeonGreen
Neg AUC: Negative Area under the curve
Pos AUC: Positive Area under the curve
REGA-SIGN: REaltime G protein Activation SIGNaling
