# Supplementary material for: Systematic assessment of chemokine ligand bias at the human chemokine receptor CXCR2 indicates G protein bias over β-arrestin recruitment and receptor internalization
Source: Cell Commun Signal. 2024 Jan 17;22:43. doi: 10.1186/s12964-023-01460-2 (PMC10795402; doi:10.1186/s12964-023-01460-2)
Supplement: Supplementary file 1 — Additional file 1: Figure S1. CXCL8AF647 titration. Figure S2. G protein activation by CXCR2 upon stimulation with its endogenous chemokine ligands pre-treated with CXCR2 inhibitor or vehicle. [file 12964_2023_1460_MOESM1_ESM.docx]

**Systematic assessment of chemokine ligand bias at the human chemokine receptor CXCR2 indicates G protein bias over β-arrestin recruitment and receptor internalization**

Katrijn Boon^1^, Nathan Vanalken^1^, Martyna Szpakowska^2^, Andy Chevigné^2^, Dominique Schols^1^, Tom Van Loy^1^*

^1^KU Leuven, Department of Microbiology, Immunology and Transplantation, Rega Institute for Medical Research, Laboratory of Virology and Chemotherapy, B-3000 Leuven, Belgium.

^2^Department of Infection and Immunity, Immuno-Pharmacology and Interactomics, Luxembourg Institute of Health (LIH), Esch-sur-Alzette, Luxembourg

*corresponding author


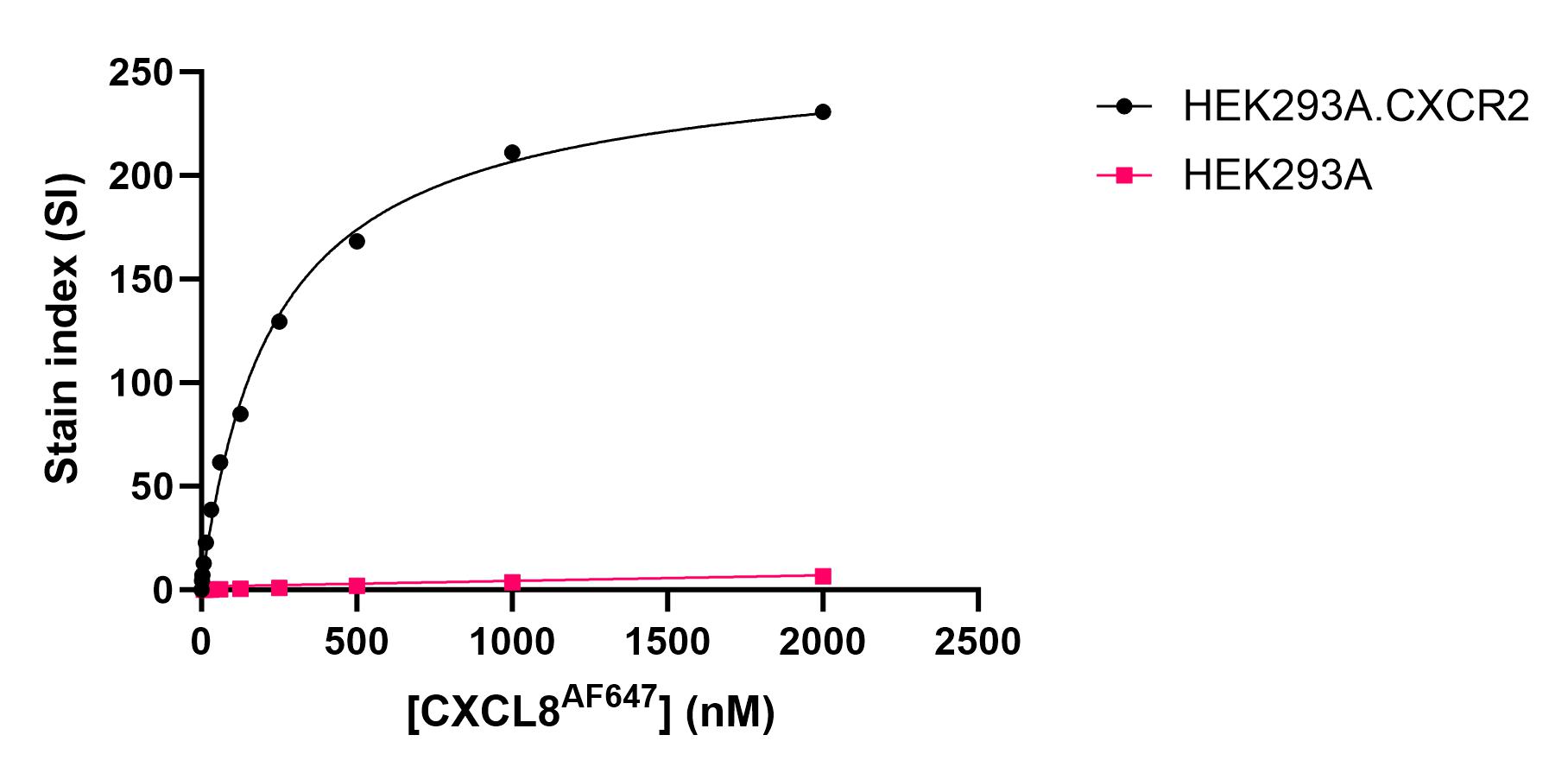


**Figure S1:** **CXCL8^AF647^ titration.** Concentration-response curves showing CXCL8^AF647^ binding towards HEK293A.CXCR2 cells and HEK293A.WT cells. Kd value was determined by fitting the curve to *One Site- Total and non-specific binding* in GraphPad V9.3.1 (GraphPad Software, San Diego, CA, USA)*.* Data represents the mean stain index (SI).


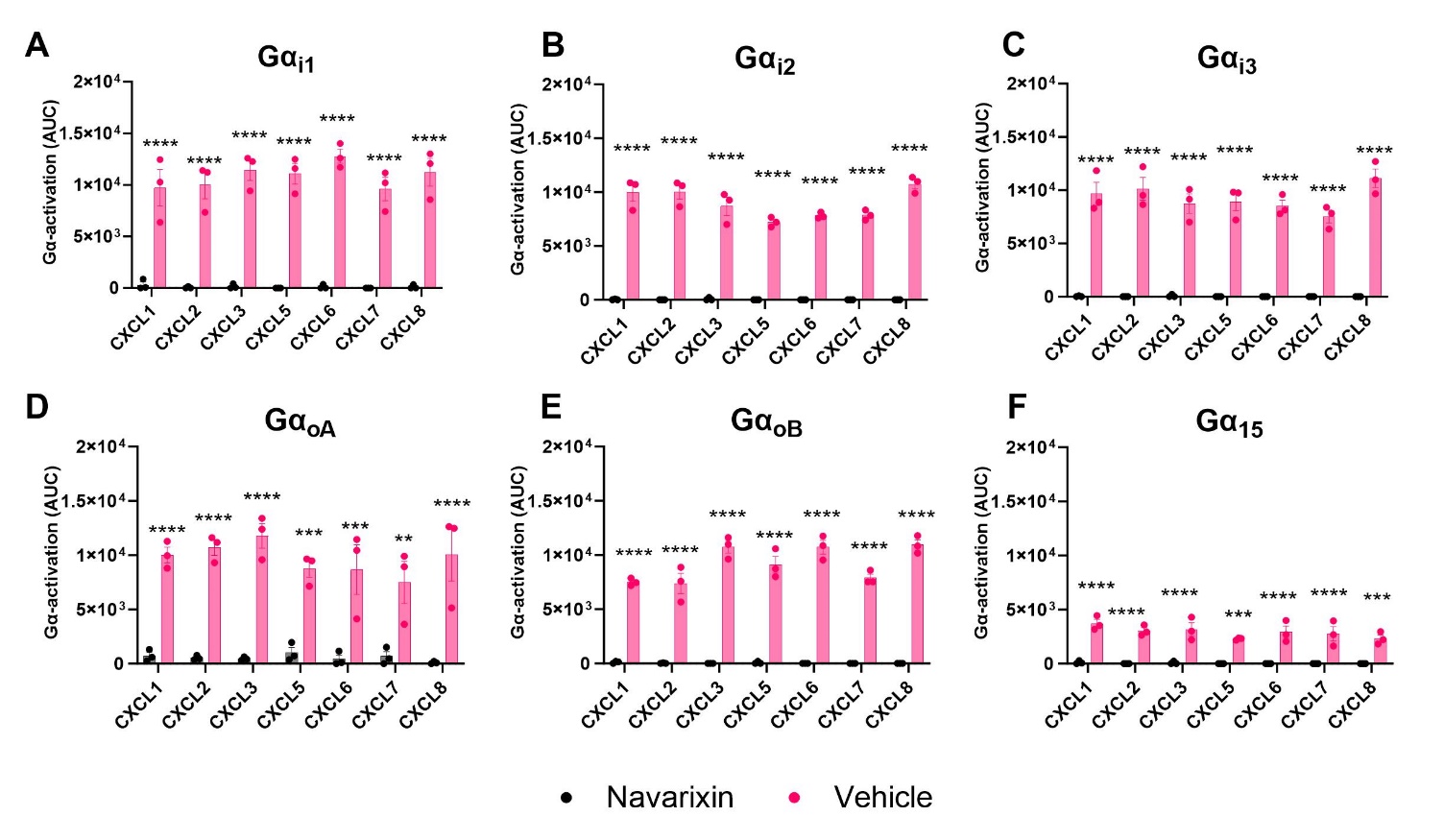


**Figure S2:** **G protein activation by CXCR2 upon stimulation with its endogenous chemokine ligands pre-treated with CXCR2 inhibitor or vehicle.** BRET signals were measured in real-time in HEK293.CXCR2 cells transfected with a REGA-SIGN donor-acceptor pair, upon stimulation with CXCL1,-2,-3,-5,-6, 7, or CXCL8 (EC_80_), in presence or absence of navarixin. Results are expressed as G protein activation using baseline-corrected Neg AUC as a read-out. Data represents the mean ± SEM of three independent experiments. Statistical significance compared to the navarixin response was calculated with two-way ANOVA followed by a Dunnet’s test: **** (P<0.0001), ***(P<0.001), **(P<0.01)
